# Subchondral tibial bone texture of conventional X-rays predicts total knee arthroplasty

**DOI:** 10.1038/s41598-022-12083-x

**Published:** 2022-05-18

**Authors:** Ahmad Almhdie-Imjabbar, Hechmi Toumi, Khaled Harrar, Antonio Pinti, Eric Lespessailles

**Affiliations:** 1 EA 4708- I3MTO Laboratory, University of Orleans, Orleans, France; 2 Translational Medicine Research Platform, PRIMMO, Regional Hospital of Orleans, Orleans, France; 3 Department of Rheumatology, Regional Hospital of Orleans, 14 Avenue de l’Hôpital, 45067 Orleans Cedex 2, France; 4 LIST Laboratory, University M’Hamed Bougara of Boumerdes, Boumerdes, Algeria; 5 DeVisu-Design, Visuel, Urbain, EA 2445, UPHF, Valenciennes, France

**Keywords:** Osteoarthritis, X-ray tomography, Predictive markers

## Abstract

Lacking disease-modifying osteoarthritis drugs (DMOADs) for knee osteoarthritis (KOA), Total Knee Arthroplasty (TKA) is often considered an important clinical outcome. Thus, it is important to determine the most relevant factors that are associated with the risk of TKA. The present study aims to develop a model based on a combination of X-ray trabecular bone texture (TBT) analysis, and clinical and radiological information to predict TKA risk in patients with or at risk of developing KOA. This study involved 4382 radiographs, obtained from the OsteoArthritis Initiative (OAI) cohort. Cases were defined as patients with TKA on at least one knee prior to the 108-month follow-up time point and controls were defined as patients who had never undergone TKA. The proposed TKA-risk prediction model, combining TBT parameters and Kellgren–Lawrence (KL) grades, was performed using logistic regression. The proposed model achieved an AUC of 0.92 (95% Confidence Interval [CI] 0.90, 0.93), while the KL model achieved an AUC of 0.86 (95% CI 0.84, 0.86; p < 0.001). This study presents a new TKA prediction model with a good performance permitting the identification of at risk patient with a good sensitivy and specificity, with a 60% increase in TKA case prediction as reflected by the recall values.

## Introduction

Osteoarthritis (OA), the most common form of arthritis, affects a substantial and increasing proportion of the population due to the combined effect of ageing and increasing overweight and obesity in the population. It is estimated that 250 million people are already affected by this leading cause of disability in older adults^1,2^. Although both non weight-bearing (hand) and weight-bearing joints such as the spine, hip, and knee are commonly affected, knee OA constitutes 85% of the global burden of OA in general^3^, and is the most frequently studied location in OA imaging research^4^. Bone metabolism and particularly subchondral bone plays a crucial role in the pathophysiology of knee osteoarthritis (KOA)^5^. Although several promising therapies have recently emerged that could reshape the overview of OA management in the near future^6^, there are still no approved agents that can be considered as validated disease-modifying osteoarthritis drugs (DMOADs)^7,8^, and consequently joint replacement surgery is often considered to be the sole effective treatment option for KOA when non-surgical management of KOA has failed. Knee replacement is considered an important clinical outcome of KOA^9,10^ and thus it is of the utmost importance to determine the most relevant factors that are associated with the risk of total knee arthroplasty (TKA)^11^.

Bone metabolism and particularly subchondral bone plays a key role in the pathophysiology of KOA^5,12^. Recently, trabecular bone texture (TBT) analysis of subchondral bone on conventional radiographs of the knee was shown to be a promising method for identifying patients at-risk of KOA progression^13–18^. An overview of the interest of TBT analysis in the assessment of KOA was recently published^19^. Applying a combined approach of new machine learning techniques and trabecular texture analysis has already been successfully used to improve the prediction of KOA progression in the Multicenter Osteoarthritis Study (MOST) and the OsteoArthritis Initiative (OAI) cohorts^20^.

The most important unresolved hurdle is identifying and classifying patients who are at risk for TKA based on bone texture analysis. In the present work, we aim to demonstrate that applying trabecular bone texture analysis to baseline knee radiographs can predict the risk of TKA in the OAI cohort. To our knowledge, no prior study has evaluated the utility of radiographic TBT analysis for predicting which individuals from a cohort of knee OA patients will undergo TKA.

## Methods

The data used in this study were obtained from the OAI cohort in patients with or at risk of developing symptomatic KOA in at least one knee. The OAI study protocol was approved by the National Institute of Arthritis and Musculoskeletal and Skin Diseases (NIAMS) and is registered on ClinicalTrials.gov as “Osteoarthritis Initiative (OAI): A Knee Health Study”, NCT#00080171. The OAI is a public access research database. The National Institute of Mental Health (NIMH) Data Archive (NDA) website (https://nda.nih.gov/oai/query-download) provides useful information on how to obtain permission to access to the OAI data. The access to the raw data used in our study was granted by the NDA Osteoarthritis Initiative permission group. The OAI study was carried out in accordance with all pertinent guidelines and regulations, and written and informed consent was obtained from participants prior to each clinical visit in the study. Details about the acquisition and grading protocols are available online at (https://nda.nih.gov/oai/study-details). The OAI is a longitudinal cohort study designed to identify biomarkers of the incidence and/or progression of KOA. Both knees of 4796 participants were studied using bilateral posteroanterior fixed-flexion knee radiography at baseline, and annually over 8 years of follow-up. The TKA outcome data were collected during 9 years. OAI participants were 45 to 79 years old at baseline, with or at risk of developing symptomatic KOA in at least one knee. At each yearly follow-up time point, OAI participants were interviewed and asked about TKA in the preceding 12 months^9^. The OAI study was approved by the institutional review boards at each OAI clinical site and the coordinating center (University of California, San Francisco) and informed consent was obtained from the participants. All research was performed in accordance with relevant guidelines/regulations and with the Declaration of Helsinki.

### Data selection

Cases were defined as patients underwent TKA on at least one knee from 12-months to 108-months follow-up time points from baseline. Controls were defined as patients who had never undergone a TKA prior to the 108-month follow-up time point. Exclusion criteria included patients about whom information on clinical covariates (CCov), namely age, gender and body mass index (BMI), and radiological Kellgren–Lawrence (KL) grades, was lacking, at baseline. Previously published studies have shown only moderate performance for predicting KOA progression when using pain and/or history of knee injury^21,22^. However, since data for WOMAC pain and history of previous injury were available for the OAI cohort, the CCov also included these additional predictors. Patients with any type of knee joint replacement reported on at least one knee at baseline were also excluded. At follow-up time points, patients with single-compartment prosthesis or non-confirmed TKA were also excluded. KL scoring was performed in the OAI using two expert readers who independently assessed each radiograph; differences were adjudicated by a group including a more senior reader^23^.

In this study, the inclusion/exclusion approach was set at baseline, and then knees were followed over time to see which ones underwent TKA. To evaluate the performance of the prediction models with respect to KOA severity, we considered two scenarios. In Scenario I, the knees of all patients (KL ≤ 4) at baseline were considered except those having previously undergone knee surgery, in accordance with the study of Leung et al.^24^. At follow-up time points, for a patient with TKA on one knee, only the knee that had undergone surgery was included. Furthermore, if both knees underwent TKA, only the first one was included. For each control patient, the knee with the most severe radiographic OA at baseline was excluded. Finally, if both knees of control patients had the same KL score, then one of them was randomly selected and the other one was excluded.

In accordance with the study of Podsiadlo et al.^25^, in Scenario II, knees with KL = 4 at baseline were excluded. In this scenario, for a patient who had undergone TKA on one knee, only the knee that had undergone TKA was included. If the included knee had a KL = 4 at baseline, both knees were excluded. If both knees underwent TKA, only the first one was included, unless the included knee had a KL = 4 at baseline, in which case this knee was excluded, and the other one was included. Both knees were excluded if they had a KL = 4 at baseline. For each control patient, the knee with the most severe radiographic OA at baseline was excluded. Here also, both knees were excluded if they had a KL = 4 at baseline. As a result, all included knees had a KL < 4 at baseline.

### Calculation of TBT parameters

A total of 16 TBT regions of interest (ROIs) were selected for each knee X-ray using an automated method^13,26^. The 16 ROIs cover the entire tibial subchondral bone structure, immediately under the medial and lateral cortical plates of the tibia. The choice of such ROIs was inspired from the work of Janvier et al.^13,27^.

For each knee X-ray, the ROI selection method consisted in identifying several anatomical points of the tibia, using the BoneFinder software (http://bone-finder.com/)^28,29^. The segmentation provided by BoneFinder is based on a machine-learning approach that has been trained on a large set of manually-annotated radiographs. The selected radiographs were not preprocessed prior to the segmentation process. Applied on different datasets, BoneFinder was able to detect the contour points of skeletal structures from 2D radiographs, in a fully automatic manner with high accuracy^30,31^. In our study, BoneFinder was used to localize the contour points of both femoral and tibial parts of the knee. In addition, BoneFinder does not need dedicated personnel or special training. In the current study, BoneFinder successfully provided correct contours of the femoral and tibial parts of all considered knees. Using BoneFinder, the identification process of the ROIs for each knee took only a few seconds.

**Figure 1:**
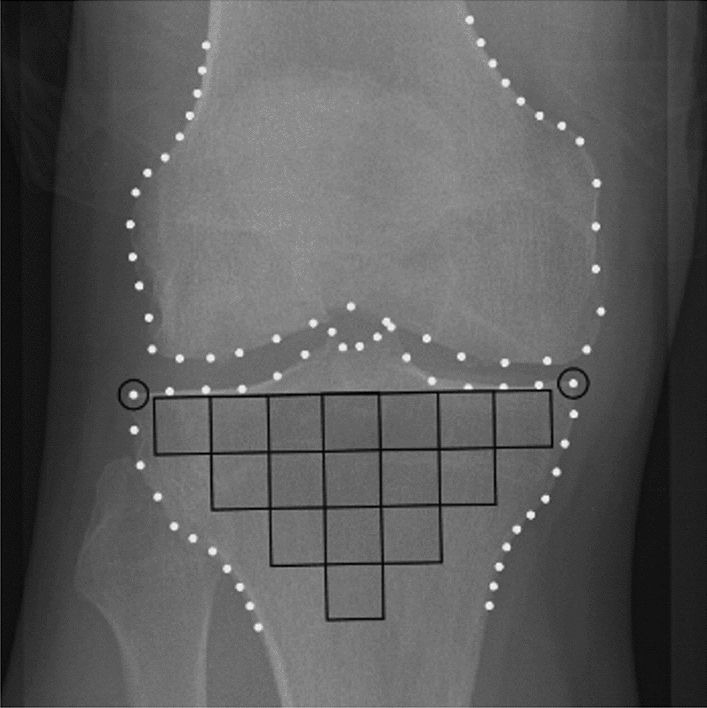
ROIs automatically selected in the tibial subchondral bone. Dots represent the femoral and tibial bone edges, delimited by BoneFinder software.

The tibial subchondral baseline was then defined as the line going between the lateral and medial extremities of the proximal tibia. To prevent periarticular osteophytes and fibular head overlay, an offset equal to 10% of the tibial subchondral baseline was applied to the horizontal positioning. Both the orientation and size of the 16-ROIs patchwork were calculated based on this line (Fig. 1). The dimension of the squared-shape ROIs was proportional to the knee width defined as the distance between the outer tibial margins.

In the present study, TBT was characterized using fractal analysis^25,32^ and the fractal parameters (H) were computed using the Variogram (VAR)^27,33^. Each ROI was quantified by means of 4 TBT variograms corresponding to the H values computed along with the two main loading directions: horizontal (*h*-direction, image lines) and vertical (*v*-direction, image columns), and for the two scales of observation: the *µ*-scale (under 400 mm) and the *m*-scale (above 600 mm). As a result, TBTp represents a vector of four TBT descriptors {H_*µv*_; H_*µh*_; H_*mv*_; H_*mh*_} computed for each of the 16 ROIs, resulting in 64 descriptors for each image, where H_*µv*_; H_*µh*_; H_*mv*_; H_*mh*_ are the microscopic horizontal, microscopic vertical, macroscopic horizontal and macroscopic vertical fractal parameters, respectively.

### Statistical analysis

**Figure 2:**
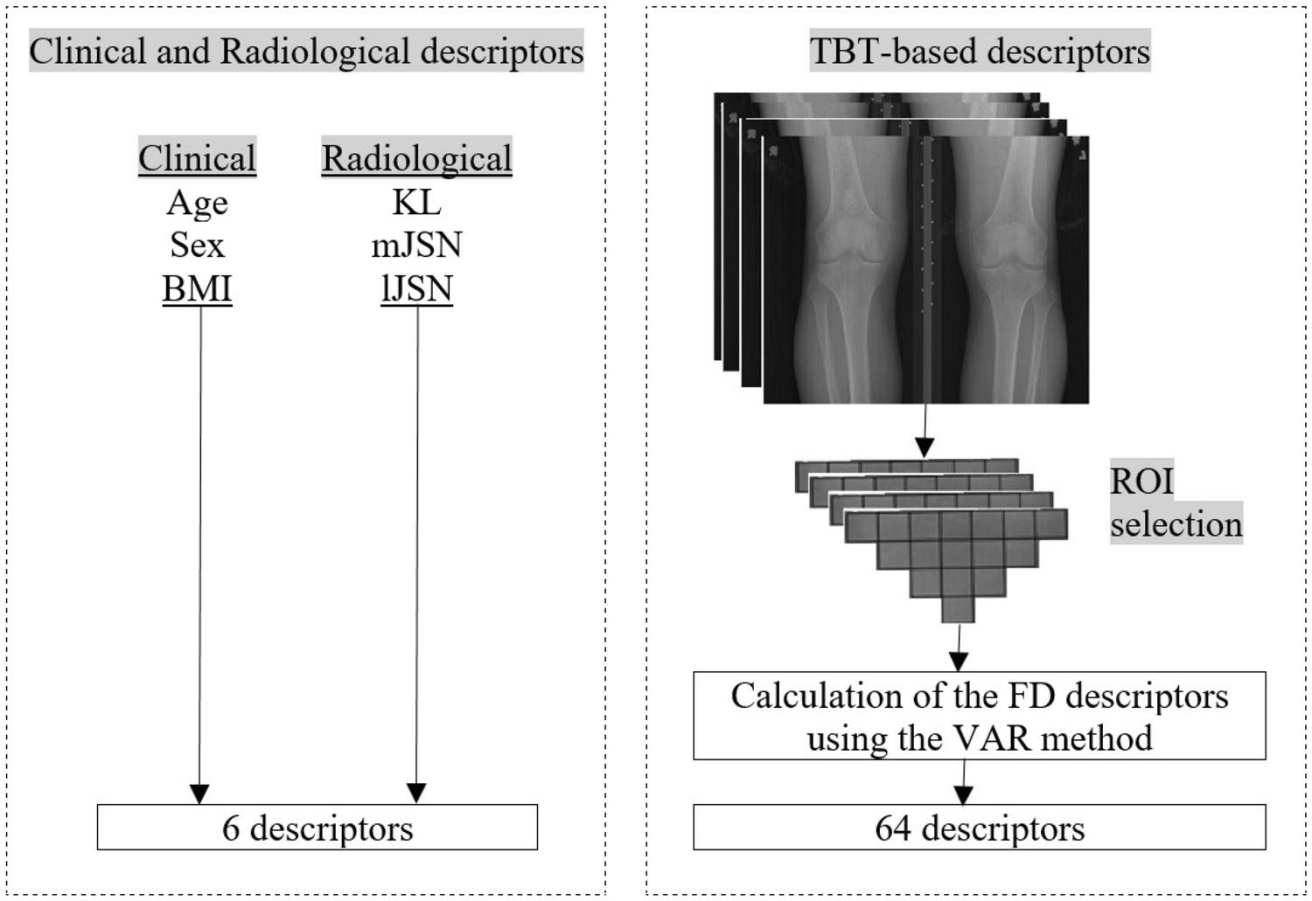
Descriptors used in the proposed model.

In this study, logistic regression was used to predict TKA risk. Logistic regression is widely used for modelling KOA progression prediction^13,16,34^ and TKA risk prediction^24^. Several statistical models were developed involving not only clinical covariates and radiological scores but also TBT-based parameters (Fig. 2):

- Model 1: CCov.
- Model 2: KL.
- Model 3: TBTp.
- Model 4: TBTp + CCov.
- Model 5: TBTp + JSN (JSN: OARSI Joint Space Narrowing in medial and JSNL compartments).
- Model 6: TBTp + KL.
- Model 7: TBTp + KL + CCov + JSNM (JSNM: medial OARSI Joint Space Narrowing).
- Model 8: TBTp + KL + CCov + JSNL(JSNL: lateral OARSI Joint Space Narrowing).

To avoid any bias in the distribution of training and validation (testing) datasets, each model was evaluated using a tenfold cross-validation repeated 300 times. The tenfold cross-validation involved randomly partitioning the given dataset into 10 equally-sized subdatasets. At each iteration, a single subdataset was used as the validation data for testing the model, and the remaining 9 subdatasets were used as training data. The whole cross-validation process was then repeated 300 times. The validation results were averaged to give a single estimate of the model's predictive performance.

**Figure 3:**
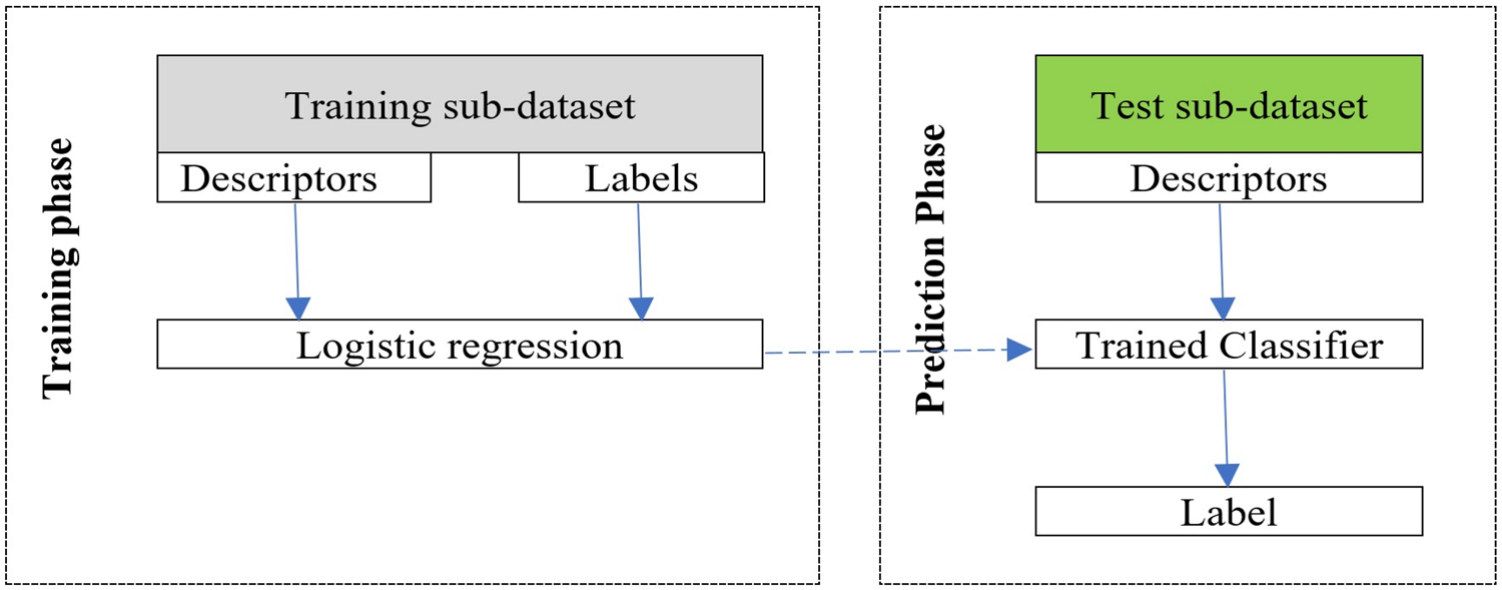
Schematic diagram of one round of machine learning prediction, repeated 300 × 10 times and then averaged.

Note that all observations were used for both training and validation, and each observation was used only once for validation. Each observation involves the descriptors of one given set of patients. The flowchart of the proposed machine learning prediction model is illustrated in Fig. 3.

The AUC was used to evaluate the global performance of the models. The model classification accuracy (ACC), i.e. the probability that a random sample is correctly classified, was also computed to investigate the relevance of the different models. An ACC is defined as the ratio of the number of correct predictions to the total number of predictions. It is expressed by:

1 $$\mathrm{ACC}=\frac{\mathrm{TP}+\mathrm{TN}}{\mathrm{TP}+\mathrm{TN}+\mathrm{FP}+\mathrm{FN}}$$

and the balanced accuracy (BA), which is a metric that is usually used to evaluate the performance of a binary classifier, is expressed by:

2 $$\mathrm{BA}=\frac{1}{2}\times \left(\frac{\mathrm{TP}}{\mathrm{TP}+\mathrm{FN}}+\frac{\mathrm{TN}}{\mathrm{TN}+\mathrm{FP}}\right)$$

where TP, FP, TN, and FN mean True Positive, False Positive, True Negative, and False Negative, respectively. Moreover, the F1 score was calculated as another performance measure of the tested models^35^. The F1 score provides a combined measure of the precision and recall of the model and defined as the harmonic mean of precision and recall. The precision refers to the positive predictive value, the percentage of correctly predicted progressors to the number of actual progressors, and the recall provides the ability of a model to predict all progressors and refers to the true positive rate or the sensitivity.

3 $$\mathrm{Precision}=\frac{\mathrm{TP}}{\mathrm{TP}+\mathrm{FP}}$$

4 $$\mathrm{Recall}=\frac{\mathrm{TP}}{\mathrm{TP}+\mathrm{FN}}$$

5 $$\mathrm{F}1=2\times \frac{\mathrm{Precision}\times \mathrm{Recall}}{\mathrm{Precision}+\mathrm{Recall}}$$

All statistical analyses were performed using the R Statistical tool^36^ (version 4.0.2, 2020-06-22) including the MASS package (Modern Applied Statistics with S, version_7.3-51.6, 2020-04-26) for stepwise Akaike Information Criterion (AIC)^37^ optimization, the Caret package (Classification And REgression Training) for the cross-validation training and the pROC for Receiver Operating Characteristic (ROC) curve comparisons, in particular partial Areas Under the Curve (pAUC). The comparison of the models’ performances was carried out with the ROC curves using the Delong method^38^. To reduce the number of parameters before training the prediction models, a backward selection of the parameters was automatically performed using the AIC as an iterative criterion. At each iteration, the AIC removes one parameter and preserves the most efficient parameter(s) to limit overfitting effects.

## Results

**Figure 4:**
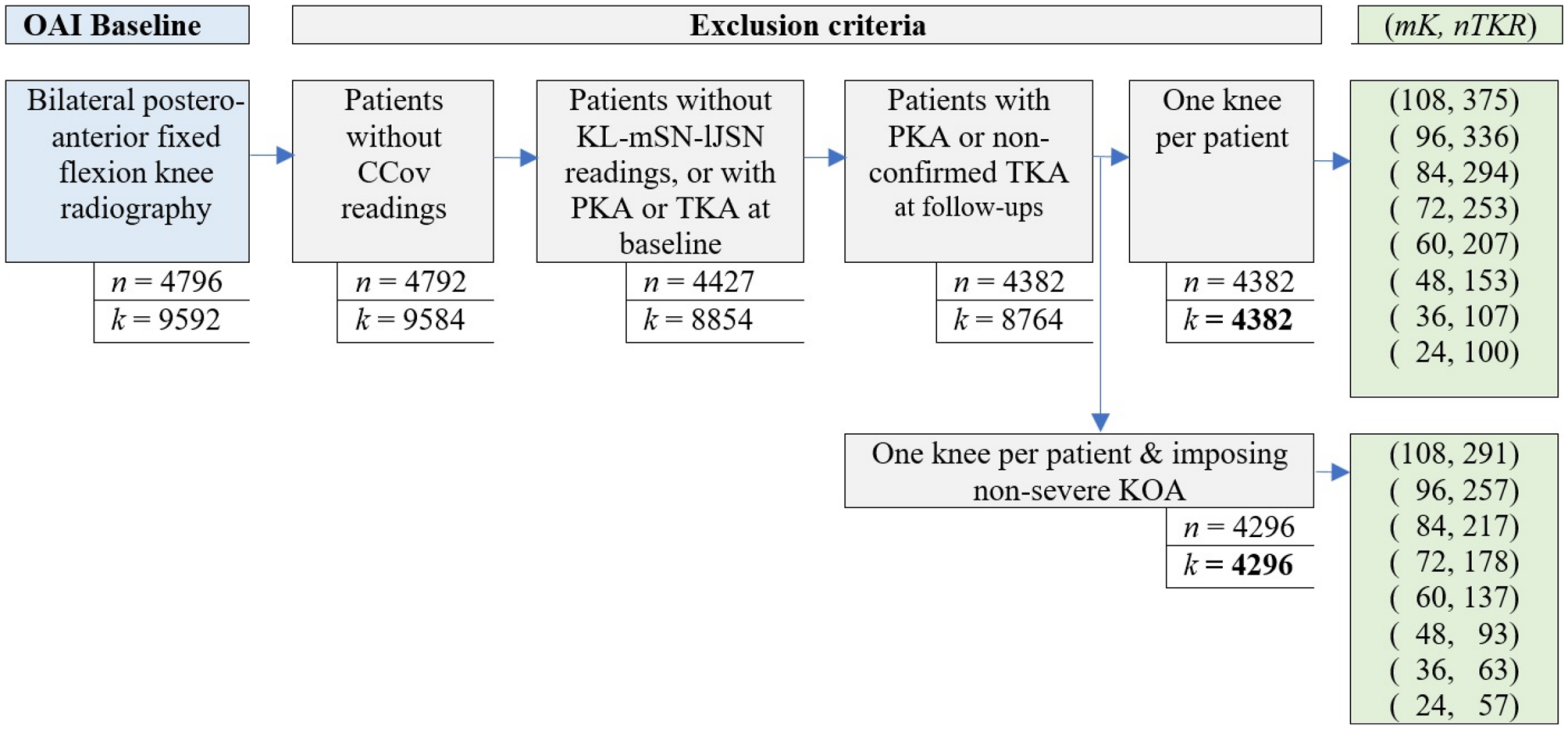
TKR-based data selection with and without imposing severe knees exclusion. *n* and *k* denote the number of patients and knees, respectively. PKA denotes partial knee arthroplasty. *nTKR* denotes the number of TKA knees prior to *mK* months’ follow-up (closest contact after TKA).

The dataset obtained for Scenario I included 4382 knees (58% women, 75% left knee) that met the inclusion criteria and were judged as eligible for this study, in which 375 knees underwent TKA on at least one knee from 12-months to 108-months follow-up time points. The dataset obtained for Scenario II included 4296 knees (58% women, 76% left knee) that were judged as eligible for this study, in which 291 knees underwent TKA prior to the 108-month follow-up. Figure 4 shows more details about our method of case–control selection, and Table 1 describes the characteristics of the two datasets.

**Table 1:** Characteristics of the datasets included in this study.

	Baseline (scenario I)			Baseline (scenario II)		
	Controls	Cases	Total	Controls	Cases	Total
N° of knees	4007	375	4382	4005	291	4296
Age (years)	$62.3(\pm 9.0)$	$62.4(\pm 8.1)$	$62.3(\pm 8.8)$	$61.0(\pm 920)$	$63.5(\pm 8.0)$	$61.0(\pm 9.2)$
BMI (kg/m^2^)	$29.4(\pm 4.6)$	$30.6(\pm 4.7)$	$29.6(\pm 4.7)$	$28.4(\pm 4.8)$	$30.0(\pm 4.9)$	$28.5(\pm 4.8)$
**Gender**						
F	57.9%	60.3%	58.1%	57.9%	65.6%	58.4%
M	42.1%	39.7%	41.9%	42.1%	34.4%	41.6%
**KL grade**						
0	2113	12	2125	2113	14	2127
1	820	22	842	820	22	842
2	834	79	913	834	91	925
3	238	153	391	238	164	402
4	2	109	111	0	0	0

Values for age and BMI are represented as mean (± standard deviation).

Figures S1 and S2, presented in supplementary files, illustrate the ROIs which offered the best predictive association with TKA based on the TBT parameters selected by the AIC algorithm. In total, 38 and 30 TBT parameters were selected in Scenario I and Scenario II, respectively.

Out of the total number of knees that underwent TKA prior to the 108-month follow-up, more than 87% had a KL ≥ 2 at baseline, while more than 73% of the control knees had a KL < 2 at baseline.

**Figure 5:**
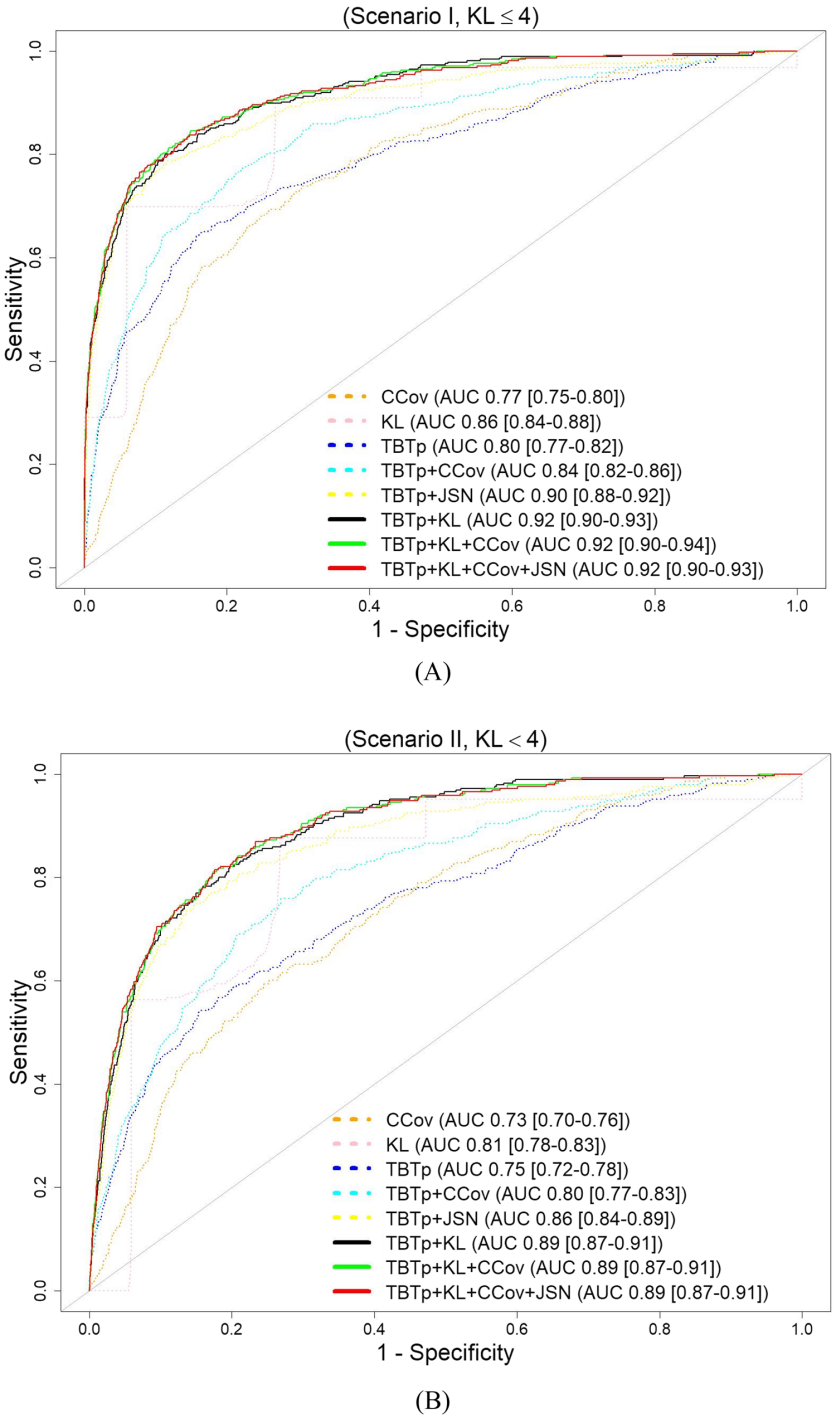
ROC curves obtained by the different TKA prediction models, using tenfold cross-validation: (**A**) using the dataset of Scenario I (0 ≤ KL ≤ 4 at baseline), (**B**) using the dataset of Scenario II (0 ≤ KL < 4 at baseline).

Figure 5 show the ROC curves obtained by the 8 prediction models listed in the previous section, using the datasets of Scenario I and Scenario II, respectively. The model based only on CCov was not strongly predictive (AUC < 0.7). Combining TBT parameters and KL grades improved the performance of the TKA-risk prediction compared to the classical reference model (KL). The TBTp-KL model achieved an AUC of 0.92 (95% Confidence Interval [CI] 0.90, 0.93), using the dataset of Scenario I and an AUC of 0.89 (95% CI 0.87, 0.91), using the dataset of Scenario II, while the KL model achieved an AUC of 0.86 (95% CI 0.84, 0.86; *P* < 0.001) using the dataset of Scenario I and an AUC of 0.81 (95% CI 0.78, 0.83; *P* < 0.001) using the dataset of Scenario II. The sensitivity of Model 1 (using only cov parameters, Scenarios I and II) and of Model 2 (using only KL scores, Scenario I) is zero, as predicting all subjects as controls. Table 2 reports the obtained F1 scores for the different tested models for both scenarios. The prediction model Model 6, combining KL and TBT, provided a better performance than the models Model 1, Model 2, and Model 3 where only one descriptor was employed as summarized in Table 2. The performance of the TBTp-KL model was not improved when including CCov, JSNM, or JSNL parameters in addition to the TBTp and KL descriptors. More statistical results are summarized in Table 2. For example, while the KL model (Model 2) provided a recall value of 0.29, the proposed model (Model 6) provided a recall value of 0.47, resulting in a 60% increase in TKA case prediction in Scenario I.

**Table 2:** Performance results of tested models in scenario I and scenario II.

Metrics	Model 1	Model 2	Model 3	Model 6	Model 7	Model 8
**Scenario I**						
Recall	0.04	0.29	0.15	0.47	0.47	0.46
Precision	0.38	0.98	0.60	0.75	0.75	0.75
Balanced accuracy	0.52	0.65	0.57	0.73	0.73	0.72
F1	0.07	0.45	0.24	0.58	0.58	0.57
AUC	0.77	0.86	0.80	0.92	0.92	0.92
**Scenario II**						
Recall	0.01	0.00	0.06	0.18	0.17	0.18
Precision	0.52	NA	0.56	0.55	0.54	0.53
Balanced accuracy	0.50	0.50	0.53	0.58	0.58	0.58
F1	0.01	NA	0.11	0.27	0.26	0.27
AUC	0.73	0.81	0.75	0.89	0.89	0.89

Where Model 1 included CCov parameters, Model 2 included KL parameters, Model 3 included TBTp parameters, Model 6 included TBTp and KL parameters, Model 7 included TBTp, KL, CCov and JSNM parameters, and Model 8 included TBTp, KL, CCov and JSNL parameters. NA refers to non-applicable values when sensitivity is zero.

## Discussion

In the current study, we explored a model based on the fractal analysis of radiographic TBT to identify a population at higher risk of TKA, even at less advanced stages of OA. Our prediction model showed that a combination of radiography-based KL scoring and TBTA provided a high level of performance as reflected by either an AUC of 0.92 (Scenario I) or an AUC of 0.89 (Scenario II).

The associations between knee arthroplasty and radiographic or clinical features of KOA have already been published^24,25,39^. The association between trabecular bone texture and knee arthroplasty was firstly studied in a limited dataset (*n* = 114 patients, 28 TKA cases)^25^. In the latter study, TBT parameters were evaluated using the Variance Orientation Transform (VOT) method. The mean fractal dimension (Texture parameter) analyzed in the medial subchondral tibial region was found to be lower in the TKA cases than in control patients. However, the study did not specify whether total or partial knee replacement, or both, was considered. Moreover, no prediction model was proposed. In the present study, we used a different TBT analysis method, (VAR), which was previously validated for the prediction of KOA progression^27^. Furthermore, we included not only the patients with severe OA (KL = 4, Scenario I), in agreement with the VOT-based study, but also the patients with non-severe OA (KL < 4, scenario II). When considering patients with (KL = 4) at baseline, the number of cases (109) was dramatically higher than the number of controls (2), contrarily to the other situations (patients with KL < 4 at baseline), as shown in Table 1. As expected, including patients with KL = 4 at baseline (Scenario I) in the training procedure provided a better prediction performance with an AUC of 0.92 than that of Scenario II (excluding patients with KL < 4 at baseline) with an AUC of 0.89.

Recently, a deep learning (DL) based approach was used for the prediction of TKA risk^24^ from the OAI cohort. In this study, the TKA outcome was defined, in line with our work, as whether a subject had undergone a TKA within 9 years or not. This binary definition of TKA does not reflect the TKA incident time. Considering time-dependent outcomes would involve the use of multinomial logistic regression instead of the binary logistic regression used in our study and it could be the subject of future investigation.

Several points, however, distinguish the present study from the previously reported one^24^. First, in the latter, radiographs were analyzed using a multitask DL approach, while in our study, a fractal-based TBT approach was used. The TBT analysis method, VAR, used in our study, has been shown to be invariant to both the quality and type of the images obtained in routine radiography^20^, whereas in DL-based methods, more attention should be paid to the variation in image quality during the training part^24^. Second, our case–control selection criteria did not impose the one-to-one case–control matching employed in their study^24^. Finally, in the current study, results related to the OARSI grades were omitted since better results of prediction were obtained with KL grades. Table 3 summarizes the main differences and similarities between the present study and the DL-based study^24^.

**Table 3:** Prediction of radiographic TKA: comparison with a recent study.

	Leung et al. (2020)^24^	Our study	
Method	Deep learning: TL & MT approaches	Fractal texture analysis: VAR method	
Cohort	Public (OAI)	Public (OAI)	
Exclusion criteria	TKA at baseline PKA Incomplete radiographic data Not one-to-one case–control matching	TKA at baseline PKA Incomplete clinical and radiographic data	
Dataset selected	728 (364 cases)	Scenario I: 4382 (375 cases) Scenario II: 4296 (291 cases)	
Prediction models	KL OARSI DL-TL-MT	KL TBT TBTp-KL	

		Scenario I	Scenario II
KL inclusion	0 ≤ KL ≤ 4	0 ≤ KL ≤ 4	0 ≤ KL ≤ 3
AUC Results	KL = 0.74 OARSI = 0.75 DL-TL-MT = 0.87	KL = 0.86 TBT = 0.80 TBTp-KL = 0.92	KL = 0.81 TBT = 0.75 TBTp-KL = 0.89

*TBT* Trabecular bone texture, *VOT* Variance Orientation Transform, *VAR* Variogram, *TKA* Total knee arthroplasty, *PKA* Partial knee arthroplasty, *AUC* Area under the ROC curve, *ROC* Receiver Operating Characteristic, *DL* Deep Learning, *TL* Transfer Learning, *MT* Multi-task, *OARSI* Osteoarthritis Research Society International, *KL* Kellgren–Lawrence.

The AUC in Scenario II was 0.89. Although significantly different from 0.92 in Scenario I (*p* = 0.022), they are both higher compared to the AUC of 0.87 (95% CI 0.85, 0.90) found in a DL-based prediction model^24^, although a direct comparison is not applicable due to the different knee-selection criteria used in both methods. The presented results highlight the potential of our prediction model for patients even with non-severe osteoarthritis (KL < 4).

Based on a dataset from the OAI cohort^18^, it was shown that both the baseline values of TBT parameters and 12–18 month changes in MRI subchondral bone texture score were significantly associated with radiographic progression at 36 months. Combining such MRI-based parameters with 2D radiographic TBT-based parameters might help to better predict TKA.

In another study^40^, more than half of the knees with no/mild radiographic OA at baseline progressed to severe radiographic OA before knee replacement during 4 years of follow-up. Patellofemoral-femoral bone marrow lesions (BMLs) and worsening pain status were often associated with knees that had no/mild radiographic OA. MRI-defined joint inflammation defined as effusion- or Hoffa-synovitis, reflecting whole joint inflammation, developed over time in a majority of knees, indicating the presence of active disease at time points close to knee replacement. It would also be interesting to evaluate the prediction capacity of a model that combines DL-based and TBT-based descriptors. Such an idea has already been investigated in the context of the prediction of KOA progression and encouraging results were found^20^. Several DL approaches have recently been proposed for the prediction of TKA^24,39,41^. The association of such approaches with radiographic TBT analysis would certainly deserve to be investigated.

Changes in subchondral bone are constitutive of the OA disease process^42^. It has been demonstrated that the subchondral bone is intimately related to the structure and function of the cartilage^43^. Subchondral bone changes in its structure and thus in its biomechanical properties interfere with the interaction between the cartilage and the subchondral bone during mechanical loading^44,45^. Distribution of local stress in the subchondral bone is part of the parameters leading to the occurrence and development of the OA process as it has been demonstrated after changes in the joint’s biomechanical environment following anterior cruciate ligament injury^46^ or associated with abnormal knee alignment^47^. Subchondral bone structure and texture analyses can thus be considered as relevant tools to reflect bone remodeling changes induced by the osteoarthritis disease process^19^.

We have considered the TKA as a relevant outcome of KOA progression, as it has been proposed by the researchers interested in KOA related studies^24,25,39,41,48,49^. However, since the occurrence of TKA may vary by patient groups, within a country or different countries, it could be better in the next future to investigate another potential outcome measure as the “End Stage Knee Osteoarthritis” score proposed by Tufts Medical Center, and accepted to be included into the Center for Drug Evaluation and Research (CDER) Biomarker Qualification Program (BQP) in 2019^50,51^, which relies on a composite of radiographic disease severity, knee pain, knee function, knee mobility, and knee instability. Despite the inherent variability in the decision to undergo TKA, the development of a predictive model is a relevant task in the aim ofaiming at improving our the current strategies to reduce the significant burden of TKA. As we lackIn the absence of approved disease modifying therapy, TKA has been considered as a relevant proxy of the end stage of KOA and an important clinical outcome in KOA^9^, even in a recently published work based on data from the OAI^52^. However, we alsothere is a lack of consensual medical indications for TKA, and with the arrival of potential new DMOADs, that target the structural course of OA disease, an earlier identification of KOA progressors will permit targeted interventions in high riskhigh-risk groups.

The limitations of this study include the non-inclusion of several patient-reported outcomes such as stiffness, quality of life, and disability in the proposed model. Clinical decision-making in TKA is a complex process, thus considering undergoing a TKA within 9 years as a single clinical outcome measure can also be a limitation of the present study. Including decision criteria of TKA eligibility (i.e., radiographic severity^53,54^, functional limitations^53^, age^54^, pain^53–55^, history of arthroscopy^55^) would help to improve the implementation and performance of proposed TKA prediction models.

Our study has still some other limitations. Only clinical covariates and radiological scores collected at baseline were included in the proposed model. The variations in these readings during the subsequent follow-ups might improve the prediction performance of our proposed model. We investigated the TBT changes in the tibial subchondral bone of the knee. More studies including the femoral zone might also be conducted to investigate the potential additional information present in the femoral subchondral bone. The comparison with the work of Leung et al.^24^ should ideally be conducted using the same set of radiographs. However, we did not have access to the reference of their selected dataset, thereby, leaving a more systematic comparison to future studies. The association between TKA and both 3D MRI bone texture^56^ or shape^57^ has recently been evaluated using data from the OAI^41,56,57^, in which features from the femoral region were also included, in addition to those from the tibial region.

The radiographs of selected patients were all acquired with a fixed flexion protocol. Therefore, our results may not applied to a study that uses a different imaging protocol. A further limitation of the dataset is that, despite the fairly large sample size, there are a very limited number of TKA cases. Only 100 such cases existed in the entire OAI for the time interval of 2 years. Consequently time-to-event experiments were not carried out in this study. Finaly, since the occurrence of TKA may vary by patient groups, with a country or different countries, it might be better to investigate another potential outcome measure as the “End Stage Knee Osteoarthritis” (esKOA) Score proposed by Tufts Medical Center, and accepted by the FDA in 2019^50,51^, which relies on a composite of radiographic disease severity, knee pain, knee function, knee mobility, and knee instability.

Our study had several strengths. The data used in our study was obtained from a validated and well-structured research database (OAI cohort) that represents a large well-phenotyped US population. In this paper, we present the largest study, in terms of number of predicted events (n = 375 in Scenario I and 291 in Scenario II) that investigated the role of TBT parameters as TKA risk predictors. Furthermore, a number of clinical predictors were included in our tested models such as history of knee injury, WOMAC pain, age, gender and BMI, in addition to radiographic disease stage (KL and two OARSI parameters: JSNM and JSNL). Another significant strength of our study is the performance analysis of several prediction models with strong validated performance metrics such as precision, recall (sensitivity), accuracy and F1 metrics^35^. In addition, technically, the ROIs used for our TBT analysis were segmented in a fully automated manner.

The clinical relevance of this study includes the feasibility of integrating the proposed model in a standard clinical routine. The used image processing tools are simple, fully automatic and based on radiography which is widely available and relatively not expensive. Furthermore, while the traditional KL model was capable to predict correctly less than 30% of total number of cases in Scenario I, and was completely incapable to predict any cases in Scenario II, the proposed KL + TBT model was capable to predict correctly 47% and 18% of total number of cases in Scenario I and Scenario II, respectively, Table 2. Suggesting radiographic TBT analysis may have a strong role in TKA risk screening, considering the lower cost and more readily implementable of radiography in primary care practice than MRI-based analysis.

Targeting high-risk individuals to prevent the progression of structural KOA might be possible with the advent of new algorithms based on multiple clinical, biologic and imaging biomarkers such as radiographic-based TBT parameters and MRI-derived features.

Our proposed prediction model performed well in the OAI cohort, in which a large number of participants were included. This type of modeling is often criticized for its inability to reproduce the same effectiveness in another sample. Although not with the same clinical endpoint (progression of the KOA disease), we demonstrated that the TBT-based models performed well not only when trained and tested on the same cohort, but also when trained on one cohort (OAI or MOST) and tested on the other one (MOST or OAI)^20^. As previously mentioned, the number of TBT parameters was optimized using the AIC.

## Conclusions

The model presented in this study, involving TBT analysis of conventional knee radiographs associated with radiological scoring (KL scores), improves the long-term prediction of TKA risk in patients with KOA.

The clinical decision-making process in TKA is complex^53^. Early detection and assessment of KOA prognostic factors are crucial for developing management and treatments that aim at preventing irreversible damage to the knee joint leading to arthroplasty. Identifying the most at-risk patients who may undergo total knee arthroplasty (TKA) could be the basis of a more aggressive therapeutic approach to prevent KOA progression.

## Supplementary Information

Supplementary Figures.
