# Supplementary material for: Subchondral tibial bone texture of conventional X-rays predicts total knee arthroplasty
Source: Sci Rep. 2022 May 18;12:8327. doi: 10.1038/s41598-022-12083-x (PMC9117303; doi:10.1038/s41598-022-12083-x)
Supplement: Supplementary file 1 — Supplementary Figures. [file 41598_2022_12083_MOESM1_ESM.pdf]

Title:

Subchondral tibial bone texture of conventional X-Rays predicts total knee arthroplasty

Authors:

Ahmad ALMHDIE-IMJABBAR[1,2], Hechmi TOUMI[1,2,3], Khaled HARRAR[4], Antonio PINTI[1,5], Eric LESPESSAILLES[1,2,3]

Affiliations:

[1]EA 4708- I3MTO Laboratory, University of Orleans, Orleans, France
[2]Translational Medicine Research Platform, PRIMMO, Regional Hospital of Orleans, France
[3]Department of Rheumatology, Regional Hospital of Orleans, France
[4]LIST Laboratory, University M'Hamed Bougara of Boumerdes, Algeria
[5]DeVisu-Design, Visuel, Urbain, EA 2445, UPHF, Valenciennes, France

E-mail addresses:

Ahmad ALMHDIE-IMJABBAR, (ahmad.almhdie@univ-orleans.fr), Hechmi TOUMI (hechmi.toumi@univ-orleans.fr), Khaled HARRAR (khaled.harrar@univ-boumerdes.dz), Antonio PINTI (antonio.pinti@uphf.fr), Eric LESPESSAILLES (eric.lespessailles@chr-orleans.fr)

Corresponding author:

Eric Lespessailles, Regional Hospital of Orleans, 14 avenue de l’Hôpital, 45067, Orleans Cedex 2, France

Supplementary Material

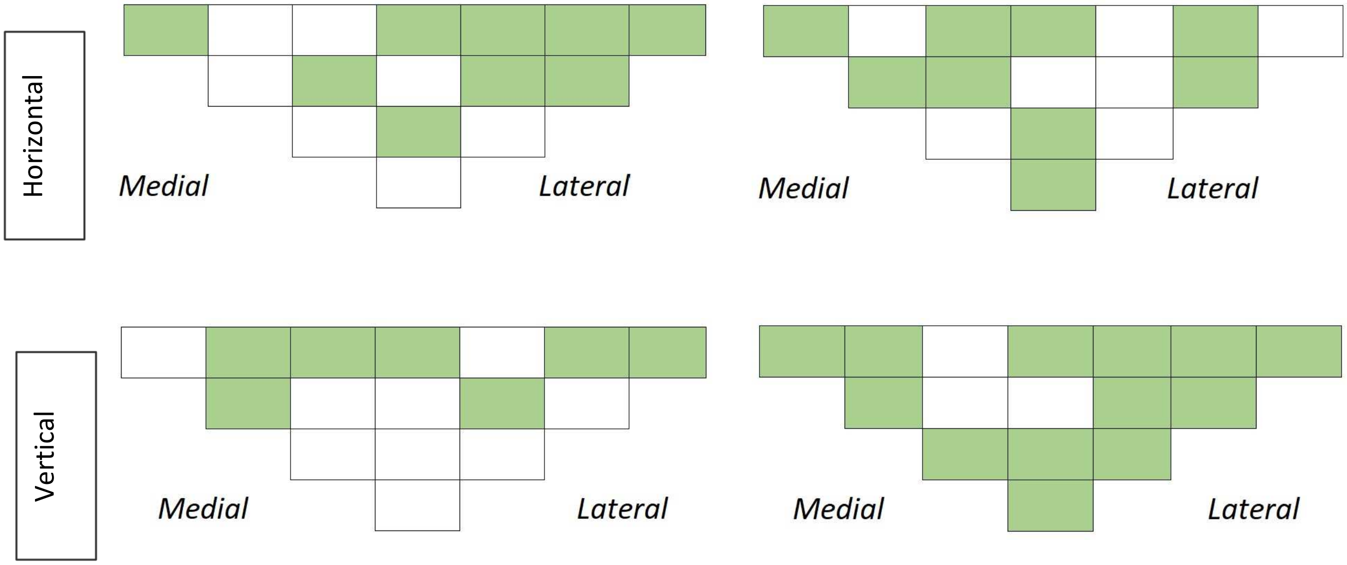

Figure S1: The most efficient TBT parameters chosen by the AIC algorithm for Scenario I. Parameters calculated in the micro- (left) and milli- (right) scales, in the horizontal (top) and vertical (bottom) directions

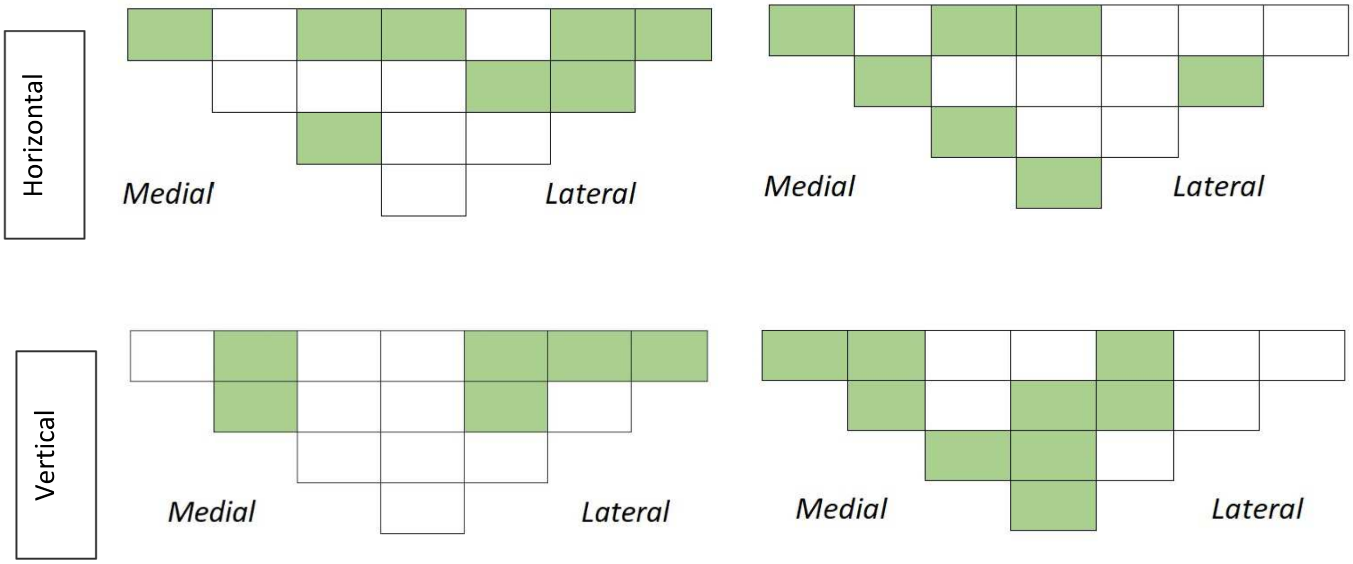

Figure S2: The most efficient TBT parameters chosen by the AIC algorithm for Scenario II. Parameters calculated in the micro- (left) and milli- (right) scales, in the horizontal (top) and vertical (bottom) directions
